# Awareness of oral health, hygiene practices, and chemotherapy-related oral manifestations among parents of pediatric oncology patients

**DOI:** 10.1038/s41405-026-00448-7

**Published:** 2026-06-03

**Authors:** Vanshika Gandhi, Mansi Pal, Vathsala Patil, Radhika Garg, Deepak Kumar Singhal, Archana M V, Adarsh Kudva, Yogesh Chhaparwal, Nanditha Sujir

**Affiliations:** 1 Department of Oral Medicine and Radiology, Manipal College of Dental Sciences, Manipal Academy of Higher Education, Manipal, Karnataka India; 2 Department of Public Health Dentistry, Manipal College of Dental Sciences, Manipal Academy of Higher Education Manipal, Manipal, Karnataka India; 3 Department of Pediatric Haematology and Oncology, Kasturba Medical College, Manipal Academy of Higher Education Manipal, Manipal, Karnataka India; 4 Department of Oral and Maxillofacial Surgery, Manipal College of Dental Sciences, Manipal Academy of Higher Education Manipal, Manipal, Karnataka India; 5 Department of Oral Medicine and Radiology, Manipal College of Dental Sciences, Mangalore, Manipal Academy of Higher Education, Manipal, Karnataka India

**Keywords:** Oral hygiene, Oral cancer detection

## Abstract

**Background:**

Pediatric oncology patients are highly susceptible to oral complications during chemotherapy. Awareness amongst parents and oral hygiene practices play a crucial role in preventing and managing these issues.

**Aim:**

To assess parental knowledge and awareness about the oral hygiene practices and reported oral manifestations associated with chemotherapy in pediatric oncology patients.

**Materials and methods:**

This was a questionnaire-based cross-sectional study conducted in the Department of Pediatric Oncology over one year. Parents of 66 children aged 0–18 years completed a structured questionnaire covering dental care utilization, oral hygiene practices, awareness regarding oral health, pre-treatment dental status, and self-reported oral side effects after therapy.

**Results:**

Only 25.7% of children had ever visited a dentist, and 12.1% underwent dental evaluation before oncology treatment. While 57.6% brushed once daily, 51.5% required assistance, and 63.6% consumed snacks between meals. Parental awareness regarding the impact of diet and oral health on general health was low (30.3%). More than half of parents (57.6%) reported oral changes after the initiation of therapy, with oral ulcers (40.9%), difficulty eating (30.3%), and taste alteration (27.3%) being most common. Despite these symptoms, only 3% underwent dental treatment post-therapy initiation.

**Conclusion:**

The study reveals inadequate dental care utilization, suboptimal oral hygiene practices, and lack of awareness amongst parents of pediatric oncology patients. Routine dental screening, structured oral hygiene education, and multidisciplinary collaboration is essential to minimize oral complications and improve oral health during chemotherapy.

## Introduction

An estimate of 400,000 children and adolescents between the age of 0 to19 years develop cancer. Leukemia, brain tumors, lymphomas, and solid tumors particularly neuroblastoma and Wilms tumor constitute most childhood cancers. While five-year survival rates in high-income countries exceed 80%, they fall below 30% for children diagnosed in lower-middle-income countries [1]. Chemotherapy and radiotherapy are the modalities that are often opted as a treatment of choice for treating these pediatric malignancies. Even though these treatment modalities have improved the chances of survival for these young patients, their non-selective cytotoxic effects pose them at higher risk for various adverse effects. Oral health complications represent a significant but frequently disregarded aspect of pediatric oncology care [2].

These children frequently complain of mucosal pain, ulcers, and trouble eating, which adds to their existing disease burden. Due to the oral mucosal sensitivity to chemotherapy medications, these patients frequently experience complications such mucositis, xerostomia, and fungal infections [3]. Chemotherapy and craniofacial irradiation can also lead to long-term dental anomalies like enamel hypoplasia, microdontia, root stunting, and delayed tooth eruption by disrupting odontogenesis [4]. The burden of oral diseases is further increased by taste changes, dysphagia, oral pain, and difficulty in maintaining regular oral hygiene, all of which have a detrimental impact on nutrition and quality of life [1]. When taken as a whole, these oral problems raise the risk of systemic infections and interfere with cancer treatment compliance, underscoring the significance of early oral management and preventative dental care for pediatric oncology patients [5].

Parents of these children are so preoccupied with their childs detrimental condition, that they often tend to ignore the oral health and hygiene of their children. Dental care is also severely hampered due to parents inadequate knowledge regarding their eating habits and its impact on oral health and also about the side effects of the therapy [6–8]. This study aims to assess the awareness amongst the parents of pediatric oncology patients who are receiving chemotherapy regarding their knowledge and awareness about their child’s oral health, oral hygiene habits and oral manifestations caused due to the treatment.

## Materials and methods

A questionnaire-based survey was conducted in the Department of Pediatric Oncology, Kasturba Hospital, Manipal over a period of one year from May 2024 to May 2025. Prior to the study, approval was obtained from the Institutional Ethics Committee of Kasturba Medical College, Manipal IEC2/359. The purpose and voluntary nature of the study were explained and written informed consent was obtained from each participant.

This was a time-bound, questionnaire-based study conducted among parents of pediatric patients (0–18 years) undergoing chemotherapy. A convenience sampling method was adopted due to the clinical condition and limited accessibility of patients undergoing active treatment. The study focused on assessing parental awareness regarding oral hygiene and dental care, irrespective of the type of malignancy or treatment protocol. Parents who agreed to participate and gave their consent,  were included. Those who were unwilling to answer the survey questionnaire were excluded. During the study period, a total of 90 eligible parents were approached to participate in the survey, of whom 66 consented and were included in the study, while 24 declined to participate. The questionnaire consisted of 5 domains:

1. Utilization of dental care
2. Child’s oral hygiene practices
3. Parental knowledge and awareness about oral health and hygiene 
4. Oral health status before initiation of chemotherapy
5. Self-reported oral side effects after the initiation of chemotherapy

The questionnaire was filled by the researcher through interview method with the parents during their waiting hours. The questionnaire was administered in both English and Kannada to ensure better comprehension among participants. Responses were recorded anonymously to maintain confidentiality.

### Statistical analysis

Data were entered in the Excel sheet and analysed statistically using SPSS (Statistical Package for the Social Sciences version 23, IBM Corp, Armonk, N.Y, USA). Descriptive analyses was done for the frequency and percentage distribution of the responses of study participants.

## Results

A total of 66 parents aged between 24 and 70 years (mean ± SD: 36.4 ± 3.5) participated in the study. Table 1. describes the oral hygiene practices reported by the parents of 66 pediatric oncology patients. The study results showed that the majority of the children brushed their teeth by themselves (69.7%). In terms of brushing frequency, only 37.8% (*n* = 25) of the them brushed their teeth twice a day, which is the best recommended practice, while the majority, 57.6% (*n* = 38), brushed their teeth once a day. Moreover, 42.4% (*n* = 28) of the children used antibiotic mouthwash, while 57.6% (*n* = 38) did not use mouthwash at all.

**Table 1:** Oral hygiene practices of the pediatric oncology patients during the therapy as reported by the parent.

Questions	Responses	Frequency, N (%)
Who is brushing the child’s teeth?	Child by himself/herself	46 (69.7%)
	Parents	20 (30.3%)
Whether using antibiotic mouthwash?	Yes	28 (42.4%)
	No	38 (57.6%)
Whether child need assistance during the brushing?	Yes	34 (51.5%)
	No	32 (48.5%)
Whether the child is restricting snacking in between meals?	Yes	24 (36.4%)
	No	42 (63.6%)
How many times child brushes his/her teeth?	Once a day	38 (57.6%)
	Twice a day	25 (37.8%)
	Not brushing regularly	3 (4.6%)
What are the oral hygiene measures used by the child	Toothbrush and toothpaste	66 (100%)
	Toothbrush and toothpowder	0 (0%)
	Charcoal	0 (0%)
	others	0 (0%)

Table 2 reflects the overall utilization of dental services till date, irrespective of their cancer diagnosis. It shows that use of dental facility among the children is remarkably low, with only 25.76% (*n* = 17) having visited a dentist, which is a low level of engagement with professional dental care services. Of those who had visited a dentist, 16.66% (*n* = 11) had visited for routine check-ups and cleaning, whereas a meagre 3.03% (*n* = 2) visited due to dental problems. In the past 6 months, only 10.6% (*n* = 7) of the children had visited a dentist. This low level of professional dental assessment is an indicator of a lack of access to preventive dental services and early intervention, which could increase the risk of undiagnosed and untreated dental problems.

**Table 2:** Utilization of dental care services before the initiation chemotherapy.

Questions	Responses	Frequency, N (%)
Has the child ever visited a dentist	Yes	17 (25.76%)
	No	49 (74.24%)
What was the purpose of dental visit?	General checkup and Tooth cleaning	11 (16.66%)
	Filling of the tooth	3 (4.5%)
	Tooth Pain	3 (4.5%)
When did the last child visit the dentist?	Less than 6 months	7 (10.6%)
	Within last 6–12 months	3 (4.5%)
	More than 1 year ago	7 (10.6%)

Further, more than half of the parents (59.1%) were unaware that eating habits could affect the child’s oral health; hence, frequent snacking was commonly reported. They (54.5%) reported to have 1–2 times of snacking in between meals, and most of the parents (69.7%) were unaware that the oral health of the children could interfere with their general health. Table 3 describes the parental knowledge and awareness regarding eating habits and oral hygiene in pediatric oncology patients.

**Table 3:** Parental knowledge and awareness regarding eating habits and oral hygiene of in pediatric oncology patients.

Questions	Responses	Frequency, N (%)
Do you believe that eating habits can interfere with child’s oral health?	Yes	20 (30.3%)
	No	46 (69.7%)
Are you restricting frequent snacking in between meals??	Yes	27 (40.9%)
	No	39 (59.1%)
How many times does a child eat between meals?	0	12 (18.2%)
	1–2 times a day	36 (54.5%)
	3–4 times a day	18 (27.3%)
Do you believe that oral health of your child can interfere with general health and the current ongoing treatment?	Yes	20 (30.3%)
	No	46 (69.7%)

**Fig. 1:**
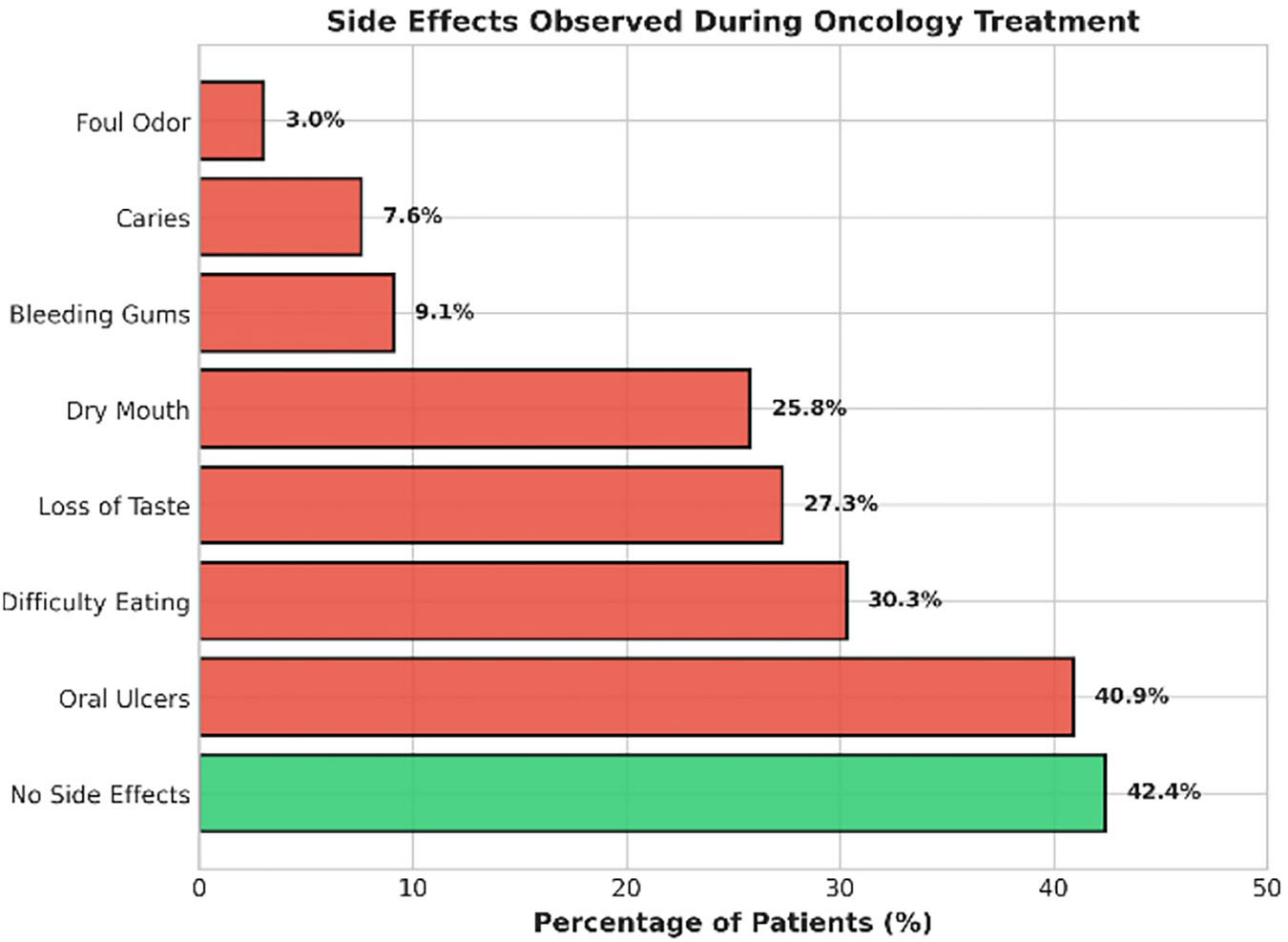
Depicting the various self-reported oral manifestations during the therapy. The red bars indicate the various oral findings reported. The green bar represents the amount of people who had no oral findings.

Maximum children (93.4%) in our study group did not have any oral complaints before the commencement of their therapy. However, 40.9% of them reported noticing oral ulcers and other oral effects, such as difficulty in eating (30.3%), loss of taste (27.3%), dryness of mouth (25.8%), bleeding gums (9.1%), occurrence of new caries (7.6%), and foul odor (3.03%) during the therapy and most of them did not seek oral health care (Fig. 1).

Even though many of them had reported having received oral hygiene instructions (60.6%), and were following these instructions, 51.5% of parents reported being unaware of the effects of therapy. 87.9% of them did not undergo dental evaluation before oncology treatment. [Tables 4 and 5].

**Table 4:** Dental consideration before oncology treatment.

Questions	Responses	Frequency, N (%)
Does the child have any dental problem before starting the oncology treatment	Yes	4 (6.06%)
	No	62 (93.94%)
What was the dental problem?	Tooth Pain	3 (4.55%)
	Bleeding gums	1 (1.52%)
Was the childs oral health status evaluated by a dentist/ dental professional prior to undergoing oncology treatment	Yes	8 (12.1%)
	No	58 (87.9%)
Did the child receive oral hygiene instructions before commencement of therapy?	Yes	40 (60.6%)
	No	26 (39.4%)
Does your child follow these oral hygiene instructions daily?	Yes	40 (60.6%)
	No	26 (39.4%)
Were you informed about the effects of therapy on oral mucosa and teeth prior to undergoing therapy?	Yes	34 (51.5%)
	No	32 (48.5%)

**Table 5:** Effects on oral health during the oncology treatment as reported by parents.

Questions	Responses	Frequency, N (%)
Did you notice any changes in the oral cavity after commencement of therapy?	Yes	38 (57.6%)
	No	28 (42.4%)
Has the child reported any oral health problems after commencement of therapy?	Yes	21 (31.8%)
	No	45 (68.2%)
What are the side effects noticed by the parents in their childs oral cavity after starting the therapy?	No side effects	28 (42.4%)
	Oral Ulcers	27 (40.9%)
	Difficulty in eating	20 (30.3%)
	Loss of taste	18 (27.3%)
	Increased thirst, dryness of mouth	17 (25.8%)
	Bleeding gums while brushing	6 (9.1%)
	Occurrence of Caries	5 (7.6%)
	Foul odor	2 (3.03%)
After starting the therapy, did the seek any oral health care for the oral problems reported by your child	Yes	2 (3.03%)
	No	64 (96.97%)

## Discussion

Oral hygiene awareness and  good oral hygiene practices play an important role in preventing oral side effects in children undergoing cancer therapy [9]. This study highlights major gap in the utilization of dental facilities, preventive measures, and parental knowledge amongst the cohort of peadiatric oncology parents. The cytotoxic effects of chemotherapy and/or radiation therapy on rapidly dividing oral epithelial cells, salivary glands, and the immune system causes various oral side effects [10]. The most common and inevitable oral complication is oral mucositis that manifests as erythema and ulceration [11]. Chemotherapy induced myelosuppression causes neutropenia and thrombocytopenia that results in increased risk of opportunistic oral infections like bacterial, fungal (candidiasis), and viral infections like herpes simplex, as well as spontaneous gingival bleeding [12]. They may also experience dryness of the mouth (xerostomia), altered salivary microbiota, reduced buffering capacity resulting in higher risk of periodontal disease and dental caries [13].

More than half of the parents (57.6%) reported noticing oral changes after the treatment initiation. The most common side effects were oral ulcers (40.9%), difficulty in eating (30.3%), loss of taste (27.3%), and xerostomia (25.8%). These findings align with the well-documented adverse effects of oncology therapy [10, 11]. However, only 3% of children received dental treatment after therapy initiation, indicating significant under-utilization of supportive dental care even when symptoms were present. Similar patterns of low dental service utilization during cancer therapy have been reported previously [14]. The significance of educating pediatric oncology parents about the different oral manifestations of chemotherapy and management strategies is implied by these findings.

Evidence supports a direct relationship between good oral hygiene and reduction in oral complications during oncology treatment. Cheng et al. reported a 38% reduction in ulcerative oral mucositis among pediatric cancer patients who followed a structured oral care protocol including gentle toothbrushing, chlorhexidine and saline rinses, compared with standard care [15]. Poor plaque control and inadequate oral care are associated with more severe mucositis, opportunistic infections, and episodes of bacteremia, which can prolong hospital stay and interrupt chemotherapy schedules and also affect the quality of life of cjhildren. However, implementation of standardized preventive protocols in pediatric leukemia patients has shown significant reduction in plaque and gingival inflammation and improve oral microbial balance over time, highlighting that sustained oral hygiene measures can modify the severity of treatment-related oral toxicity. Thus, reinforcing daily oral hygiene practices in this high‑risk group is an integral component of supportive oncology care [9, 16, 17].

Parental awareness of oral health was found to be limited in our study. Only 30.3% believed that eating habits influence their child’s oral health and believed that oral health could affect general health and ongoing cancer treatment. Although 60.6% reported receiving oral hygiene instructions before therapy, nearly 40% did not, highlighting inconsistencies in preventive counseling. According to Kubsad N and Patil S, 95% of parents were ignorant of the oral symptoms of systemic diseases, 96% about medication-related oral complications, and 96% aboutpotential dental treatment difficulties [18].

The findings of the present study suggest gaps in parental awareness regarding chemotherapy and its oral effects. This may be due to lack of communication between healthcare providers and caregivers, rather than solely lack of parental knowledge [7]. Similar communication deficiencies between healthcare providers and caregivers have also been described in pediatric oncology settings [19]. **Educational material and preventive oral health initiatives through storytelling, videos and entertainment tools can increase the parents awareness of possible oral changes brought on by anticancer treatment and increases their vigilance for oral health and its link to systemic health** [17].

Dedicated dental teams have proven very helpful in such situations. According to an Italian study by Morini et al. a dedicated dental team comprising of a dentist and two hygienists offered weekly services to a pediatric cancer unit. The continuous presence of dental specialists was much appreciated by the parents of pediatric patients. The ongoing follow-ups also facilitated prompt therapy of oral consequences like mucositis, dryness of mouth. The dental team’s actions also led to functional improvement in 65.1% of cases and pain reduction in 87.15% of cases [20]. This demonstrated the beneficial effects of dental team interventions, indicating that institutional integration of dental expertise needs to become a routine procedure in pediatric oncology.

Regarding oral hygiene practices, most of the children (69.7%) brushed their teeth by themselves, but more than half (51.5%) required assistance. Suboptimal brushing practices and inadequate supervision in medically compromised children have been previously reported [9]. 57.6% of children brushed once daily only 37.8% brushed twice daily, reflecting oral hygiene practices in a high-risk group like ours. Antibacterial mouthwash use was also limited (42.4%). Snacking control was poor, as 63.6% of children consumed snacks between meals, and only 36.4% actively restricted frequent snacking. Such behaviors may contribute to increased cariogenic risk, due to already persisting xerostomia, altered salivary flow and underlying immunocompromised state [13].

To prevent caries development dietary counseling should focus on minimizing refined sugars and acidic meals. For the early detection and treatment of oral infections, dental abnormalities, and treatment-related problems, routine dental examinations before, during, and following cancer therapy are crucial [5]. Fluoride applications should be made a part of oral care protocol for patients undergoing cancer therapy. Also, adherence to good oral hygiene routine is essential for maintaining nutrition during the ongoing therapy and also to improve the quality of life [5].

In this study, only 25.7% had visited a dentist and nearly 74% reported of no previous dental visits. The lack of dental screening was further reflected by the finding that only 12.1% of children were evaluated by a dentist before the start of chemotherapy and merely 1.5% received any intervention prior to chemotherapy. Such low rates are clinically concerning as they may further exacerbate oral complications [19].

Maintaining good oral hygiene is highly advised which includes brushing teeth gently twice a day with a soft or ultra-soft toothbrush and fluoridated toothpaste (1000–1450 ppm fluoride), which can reduce the plaque-associated gingival inflammation. During severe mucositis or thromobocytopenia tooth brushing can be replaced with oral care with foam swabs or gauze soaked in saline or sodium bicarbonate solution. Alcohol-free mouthwashes like saline or sodium bicarbonate rinses are recommended to preserve oral hygiene and mucosal comfort. To ease xerostomia and lower the risk of dental caries caused by radiation or chemotherapy, it is advised to drink frequent sips of adequate water and use sugar-free chewing gum or artificial salivary supplements [5].

Overall, the study reveals a substantial gap between the oral health needs of pediatric oncology patients and the oral preventive care they receive. The low rates of dental evaluation, limited parental awareness, and inadequate oral hygiene practices suggest that oral care is not sufficiently integrated into routine pediatric oncology care protocols. Since oral complications can compromise nutrition, systemic health, and chemotherapy compliance, strengthening multidisciplinary collaboration between oncology and dental teams is essential [5, 20, 21].

This study has certain limitations that should be acknowledged. The sample size was relatively small, as the study was conducted over a defined time period (May 2024–2025) among patients undergoing active chemotherapy. Due to the vulnerable health status of this population and treatment-related constraints, a convenience sampling method was employed, resulting in the inclusion of 66 participants. While the findings provide valuable preliminary insights into oral hygiene practices and dental care needs in cancer patients, they may not be generalizable to a broader population. Future studies with larger sample sizes and multi-center designs are recommended to validate and expand upon these findings. Furthermore, due to the limited sample size and heterogeneity of participants, the statistical analysis was restricted to descriptive methods, and inferential conclusions should be interpreted with caution.

## Conclusion

This questionnaire-based survey highlights significant gaps in dental care utilization, oral hygiene practices, and parental awareness among pediatric oncology patients undergoing chemotherapy. Despite majority of patients experiencing oral side effects very few make an effort to manage them. The findings emphasize the need for improved parentral knowledge programs, mandatory pre-therapy dental screening, routine oral hygiene counseling reinforcement, and easy access to dental care during therapy. Future studies should investigate the barriers and determinants of dental utilization in oncology settings and assess the effectiveness of structured standardized oral care protocols/ regimens on treatment outcomes.

## Data Availability

The data required to reproduce these findings are available in the manuscript or in the supplementary information. Further information can be provided upon reasonable request by the corresponding author.
